# Does a change in end-tidal carbon dioxide level predict high altitude mountain sickness?

**DOI:** 10.1016/j.heliyon.2023.e16000

**Published:** 2023-05-05

**Authors:** Josef G. Thundiyil, Alex T. Williams, Ian Little, Margaret Stutsman, Jay G. Ladde, Linda Papa

**Affiliations:** Orlando Health, Department of Emergency Medicine, United States

**Keywords:** Altitude illness, Acute mountain sickness, End tidal carbon dioxide

## Abstract

**Background:**

It is postulated that lack of hypoxic ventilatory response is a predictor for AMS. End-tidal carbon dioxide (ETCO_2_) is an accurate, noninvasive surrogate measure of ventilation.

**Objectives:**

We sought to determine if changes in baseline ETCO_2_ predicts the development of AMS.

**Methods:**

This prospective cohort study took place in three separate high-altitude hiking treks. Subjects included a convenience sample of hikers. Predictor variable was change in ETCO_2_ levels and outcome variable was AMS. Measurements of ETCO_2_ levels were obtained at the base and repeated daily at various elevations and the summit of each hike. Concurrently, hikers were scored for AMS by a trained investigator. We utilized correlation coefficients and developed a linear regression model for analysis.

**Results:**

21 subjects in 3 separate hikes participated: 10 ascended to 19,341 ft over 7 days, 6 ascended to 8900 ft in 1 day, and 4 ascended to 11,006 ft in 1 day. Mean age was 40 years, 67% were males, mean daily elevation gain was 2150 ft, and 5 hikers developed AMS. The correlation coefficients for ETCO_2_ and development of AMS were −0.46 (95%CI -0.33 to −0.57), and −0.77 (95%CI -0.71 to −0.83) for ETCO_2_ and altitude. ETCO_2_ predicted the development of symptoms better than the elevation with AUCs of 0.90 (95%CI 0.81–0.99) versus 0.64 (95%CI 0.45–0.83). An ETCO_2_ measurement of ≤22 mmHg was 100% sensitive and 60% specific for predicting AMS.

**Conclusions:**

ETCO_2_ was strongly correlated with altitude and moderately correlated with AMS and it was a better predictor than altitude.

## 1 Introduction

High altitude illnesses range from acute mountain sickness to high altitude pulmonary or cerebral edema. These illnesses are affected by several factors that alter cellular and organ function. It is postulated that an inadequate hypoxic ventilatory response (HVR) is a significant contributor to the development of acute mountain sickness [1].

Current predictors for development of altitude related illness are imperfect and do not provide accurate clinical guidance on management during ascent. These include rate of ascent, vigorous exercise, pulse oximetry [2] and previous history of mountain sickness. None of these markers provide information on the physiologic predictor of ventilation. Most of these predictors also do not provide real time dynamic information during ascent.

Our aim was to determine if ventilation predicts the development of altitude sickness. End tidal carbon dioxide is a commonly used adjunct in the hospital and prehospital setting used to measure ventilation, confirm endotracheal tube placement, and guide resuscitation. We sought to determine if ventilation as measured by end tidal carbon dioxide could predict the development of acute mountain sickness. Our secondary objectives were to determine a correlation between end tidal carbon dioxide measurements in high altitude trekkers and elevation and to determine whether end tidal carbon dioxide predicts acute mountain sickness better than altitude.

## 2 Materials and methods

This prospective cohort study was conducted during three separate high-altitude treks. These included a 7-day trek to 19,341 feet on Mt. Kilimanjaro, a single day hike to an elevation of 8900 feet on Half Dome, and a single day trek to the summit of Estes Cone of 11,006 feet. These hikes started from elevations of 7740 feet, 4300 feet, and 9405 feet, respectively.

The study subjects included a convenience sample of hikers who were already scheduled to begin these ascents. Enrollment was voluntary. A total of 21 potential participants were eligible to take part in this study. IRB approval was obtained for this study and all subjects were voluntarily consented prior to data collection.

Enrolled subjects had baseline demographic data collected including age, gender, altitude sickness medication usage, rate of ascent, and elevation at which they normally resided. The predictor variable of change in end tidal carbon dioxide (ETCO_2_) was measured by an FDA approved portable capnometer (Masimo EMMA Irvine, CA, USA). This was measured at the start and summit of each hike. For the multiple day trek, measurements were performed in the morning prior to hiking and in the evening at the completion of each day. A measurement was also taken upon reaching the summit.

The primary outcome variable was development of Acute Mountain Sickness as determined by the validated 2018 Lake Louise Acute Mountain Sickness Score (LLAMSS). A trained investigator conducted this measurement concurrently and confidentially while measuring end tidal carbon dioxide. The score was recorded on a spreadsheet throughout the course of each trek. The elevation was recorded at the time of each measurement. In the presence of headache, LLAMSS score of 3–5 is defined as mild AMS while a score of 6 or more is defined as severe AMS.

We performed linear regression analysis on the measurements to determine if a relationship exists between the predictor and outcome variables. To create these regressions, the data at each site was input into Microsoft Excel. These values were then used to create simple linear regressions for the relationship between ETCO_2_ and AMS and an exponential regression between ETCO_2_ and altitude. We used an exponential regression model for altitude as ETCO_2_ values would be expected to approach a physiologic limit for which a linear regression would not account. Data analysis was confirmed with SPSS V.22.0 (IBM, Somers, New York, USA). We calculated an ROC curve for predicting the development of AMS for ETCO_2_ and altitude.

## 3 Results

All 21 eligible subjects enrolled in this study and all successfully completed the summit on their respective hikes. All participants reside at or near sea level and arrived to starting elevation within 24 h of beginning of their respective treks. None of the subjects had significant underlying medical history including pulmonary or cardiac disease. One hundred and sixty six measurements of ETCO_2_, altitude, and LLAMSS were obtained for these subjects. Five of the hikers developed mild AMS and 1 developed severe AMS. Two subjects developed mild AMS on separate days during the trek such that there were 7 measurements of mild AMS. Eleven additional subjects developed symptoms that did not reach the definition of AMS. No subjects developed high altitude pulmonary or cerebral edema. All 11 subjects who hiked Kilimanjaro took acetazolamide and 5 also took dexamethasone. No hiker on the other treks took any medication to alleviate mountain sickness. The mean daily elevation gain was 2225 feet per day. Table 1 describes the study subjects for each hike.

**Table 1:** Characteristics of study subjects and treks.

	Kilimanjaro	Estes Cone	Half Dome	Total
Number hikers	11	4	6	21
Mean Age yrs. (Range)	43.6 (29–56)	28.8 (27–33)	39.7 (35–47)	39.7
Gender (%male)	100%	25%	33%	67%
Elevation change	11,599	1601	4600	
Starting elevation (ft)	7742	9405	4300	N/A
Ending elevation (ft)	19,341	11,006	8900	N/A
Mean change (ft)/day	1933	1601	4600	2225
Number days	6	1	1	N/A
Using meds %	100%	0	0	52%
% decadron	36%	0	0	19%
%acetazolamide	100%	0	0	52%
LLAMS score (mean)	1	4	1	1
Mild	2	1	1	4
Severe	0	1	0	1
ETCO_2_ baseline (mmHg)	28	24	34	29
Mean ETCO_2_ change at summit (mmHg)	8.5	4	7	7

**Fig. 1:**
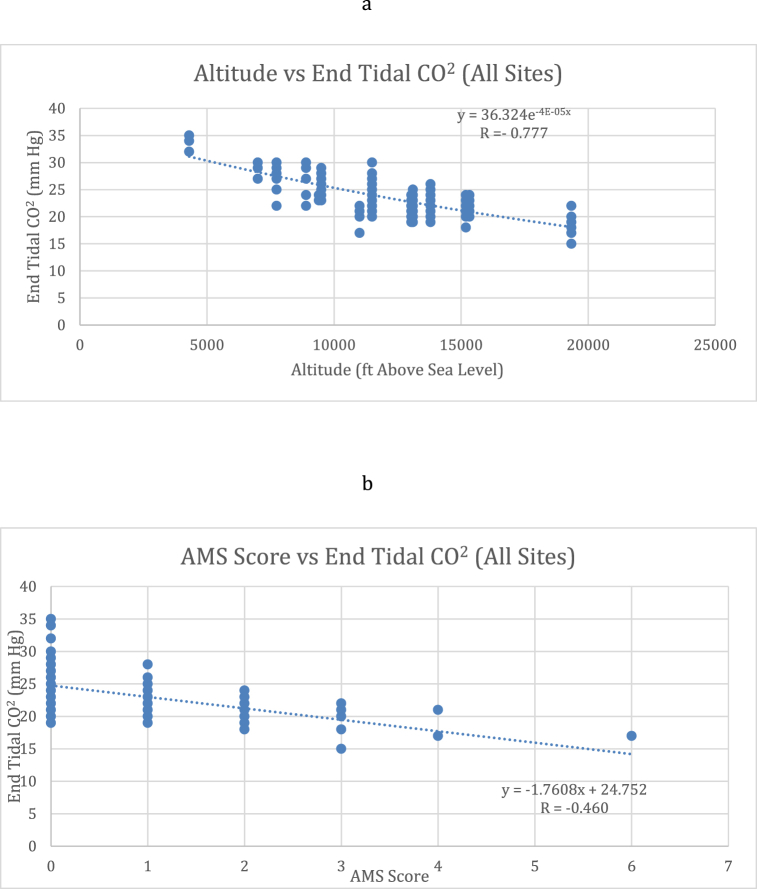
a. Exponential regression demonstrating correlation between ETCO_2_ with altitude. b. Exponential regression demonstrating correlation between ETCO_2_ with AMS.

**Fig. 2:**
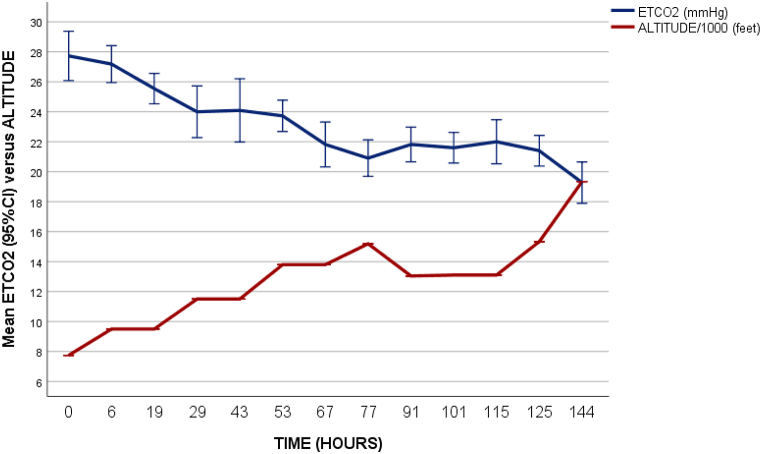
Mean ETCO_2_ measurements compared with altitude.

Mean net ETCO_2_ change was 5 mmHg (range 0–13 mmHg). Mean ETCO_2_ change was 8.2 mmHg (95%CI 5.0–11.4 mmHg) for those with AMS and 4.8 mmHg (95%CI 4.3–5.3 mmHg) for those without AMS. Fig. 1a and Fig. 1b demonstrate the exponential regression model for AMS, altitude and ETCO_2_. For all sites, the correlation coefficient (R-value) for the association between ETCO_2_ and AMS was −0.46 (95%CI -0.33 to −0.57). For each trek the R-value was 0.46 for Kilimanjaro, 0.92 for Estes Cone, and 0.55 for Half Dome. The correlation between ETCO_2_ and altitude for all sites was −0.77 (95%CI -0.71 to −0.83). For each ascent, the R-value was 0.72 for Kilimanjaro, 0.76 for Estes Cone, and 0.82 for Half Dome. Fig. 2 demonstrates the relationship between ETCO_2_ and altitude over time for all treks.

Out of 166 measurements, 46 measurements yielded an LLAMS score of 1 or greater (at least one symptom). For the LLAMS score 0, ETCO_2_ was 25 mmHg (95%CI 24–26 mmHg) (Range 19–35 mmHg), compared to a LLAMS score of 1 or greater, ETCO_2_ was 21 mmHg (95%CI 20–22 mmHg) (Range 15–28 mmHg) (P < 0.001). In this dataset, there were 9 measurements which yielded an LLAMS score of 3 or greater. For the LLAMS score <3, ETCO2 was 24 mmHg (95%CI 24–25 mmHg) (Range 18–35 mmHg), for LLAMS score of 3 or greater ETCO_2_ was 19 mmHg (95%CI 17–21 mmHg) (Range 15–22 mmHg) (P < 0.001).

**Fig. 3:**
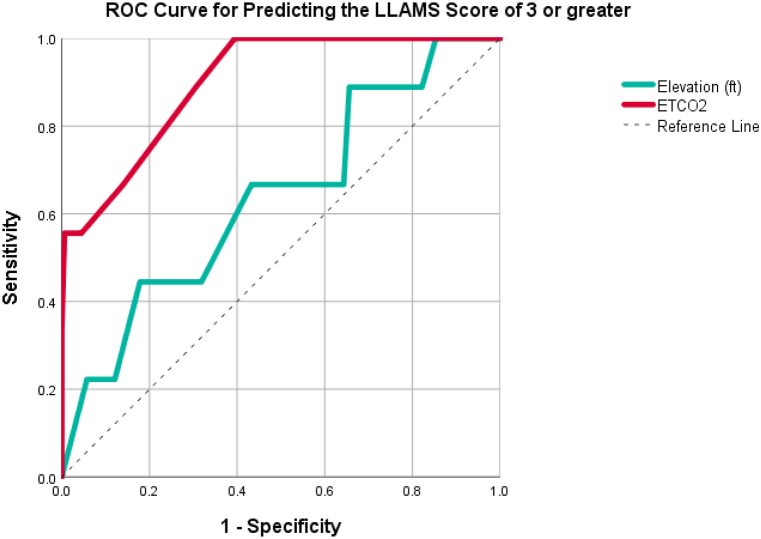
ROC curve for AMS for elevation and ETCO_2_.

The ROC curve for predicting the development of AMS symptoms with an LLAMS score of 3 or greater is shown in Fig. 3. ETCO_2_ predicted the development of symptoms better than the actual elevation with an AUC of 0.90 (95%CI 0.81–0.99) for ETCO_2_ versus an AUC of 0.64 (95%CI 0.45–0.83). For study subjects with ETCO_2_ of 22 mmHg or lower compared to those with ETCO_2_ greater than 22 mmHg, the RR of developing any symptom on the LLAMS scale was 4.32 (95%CI 2.35–7.87). Similarly, using an ETCO_2_ of 22 mmHg or lower compared to those who measured greater than 22 mmHg, the relative risk of developing at least mild AMS (LLAMSS >3) was 25.3 (95%CI 1.50–428) (P < 0.02). If we used an ETCO_2_ measurement of 22 mmHg or lower as a screening for AMS, we derived a sensitivity of 100% (95%CI 66–100%) (see Table 2).

**Table 2:** Sensitivity and specificity of using ETCO_2_ as a screening for AMS using ETCO_2_≤22 mmHg as a cutoff.

Statistic	Value	95% CI
Sensitivity	100%	66.4–100%
Specificity	60.5%	52.4–68.2%
Positive LR	2.53	2.09–3.07
PPV	12.7%	10.7–14.9%
NPV	100%	N/A

## 4 Discussion

This is the first study to evaluate the relationship between real time ETCO_2_ and development of AMS. There was a trend toward lower ETCO_2_ for those who were symptomatic. Additionally, ETCO_2_ was a better predictor for developing AMS than altitude.

Previous studies have attempted to look for noninvasive markers to predict AMS. Studies evaluating pulse oximetry readings with elevation and development of AMS have demonstrated mixed results without promising predictive value [1,3]. Additional literature has evaluated relationships with heart rate variability and development of AMS to mixed results [4]. A recent study by Burtscher et al. evaluated the change of resting minute ventilation and peripheral oxygen saturation as predictors of AMS [5]. The authors found that subjects with a smaller rate of change of minute ventilation had a higher likelihood of developing AMS. While our study does not measure minute ventilation, our results may complement the results of this study. Minute ventilation is a combination of both tidal volume and respiratory rate but does not expressly account for gas exchange. It has been postulated that a subject's inability to increase minute ventilation to account for decreases in oxygen concentration may be a contributor to development of AMS.

ETCO_2_ is a convenient, non-invasive surrogate for ventilation frequently used in the emergency department and prehospital setting. This is the first study to attempt to measure real time end tidal carbon dioxide as a surrogate marker for ventilation in hikers at various elevations. Based on the currently understood physiologic mechanisms, there was a strong negative correlation between elevation and exhaled carbon dioxide. If extrapolated, based on our exponential regression, the ETCO_2_ at the top of Mt. Everest would be approximately 11.4 mmHg. Several possible explanations exist for this finding. It is possible that this is due to the well understood respiratory alkalosis that occurs with increased altitude, but confounding factors remain.

In our study population, mean ETCO_2_ decrease was 8.2 mmHg for those with AMS and 4.8 mmHg for those without AMS. Previous studies have demonstrated variable results with end tidal carbon dioxide and altitude. Douglas et al. demonstrated an increase in ETCO_2_ with higher altitude in Cusco, Peru for a 2 day climb [6]. However, over 30% of subjects were lost to follow up in that study. Other research reveals a negative correlation between altitude and ETCO_2_ [7]. Similar to the latter study, our data demonstrated a finding of lower ETCO_2_ measurements in those that developed symptoms of AMS. The conflicting findings among studies may unveil a more complex mechanism in the development of AMS. While previous publications have indicated that a blunted HVR may predispose to AMS [8,9], other research demonstrates that the correlation between HVR and AMS is poor in low elevation dwellers during their first few days at higher elevation [10,11].

Mairburi et al. demonstrated that high altitude pulmonary edema (HAPE) susceptible patients had a blunted HVR, relatively normal alveolar ventilation during prolonged hypoxia, and a lower arterial partial pressure of carbon dioxide at high altitudes than those without HAPE susceptibility [12]. If high altitude illnesses were solely related to a relative hypoventilation predicted by blunted HVR, HAPE susceptible individuals would be expected to have higher arterial pCO_2_ than those without susceptibility. Accordingly, though a reduced ventilatory response may be seen during isocapnic HVR testing, other underlying mechanisms are present in actual high-altitude situations. These mechanisms include disturbances in oxygen utilization at the microcirculation level which lead to acid base disturbances at the cellular level [13].

ETCO_2_ levels are determined by basal metabolic rate, cardiac output, and ventilation. Changes may reflect a derangement in not only gas exchange, but also perfusion or metabolism. ETCO_2_ has previously been shown to be a highly sensitive surrogate for acid-base disturbances, correlates strongly with serum lactate, guides resuscitation, and predicts sepsis consistently [[14], [15], [16], [17], [18], [19]]. Though, capnography is most effective when assessing a clear ventilatory, metabolic or perfusion issue; mixed processes are more difficult to interpret. Although we set out to use ETCO_2_ as a marker for ventilation in this study, it is more likely that the changes reflected in ETCO_2_ amongst high altitude trekkers resulted from a mixed picture. Our results indicate that a combination of hyperventilation and a secondary metabolic process may be the culprit in inducing a further fall in ETCO_2_ in those hikers that experienced AMS. Metabolic acidosis from cellular hypoxia is a likely explanation.

Given the potential for multiple metabolic processes to affect ETCO_2_, one of the limitations of this study is the difficulty in determining whether the CO_2_ changes were entirely due to illness as opposed to baseline metabolic differences amongst hikers. To account for this, we obtained baseline measurements of ETCO_2_ for each hiker at the start of each hike. These baseline readings were very similar amongst subjects. Notably, the mean changes of ETCO_2_ demonstrated significant differences with altitude and with AMS. We do not know whether these changes reflect basal metabolic differences between subjects. One explanation is that as acidosis develops, compensatory respiratory alkalosis leads to decreased pCO_2_. As both the capacity of the renal system to produce bicarbonate and the respiratory system to compensate become overwhelmed, acidosis further develops, as does a reduction in CO_2_ and subsequently ETCO_2_. This has been demonstrated in several disease states including DKA, sepsis, acute renal failure, and drug overdose where the serum bicarbonate is directly correlated with ETCO_2_ [20]. Regardless of cause of the changes in ETCO_2_, the measurement reliably demonstrated the ability to predict the development of AMS especially as it fell below 22 mmHg. In fact, the ETCO_2_ was appreciably better than altitude in predicting AMS. Future studies should attempt to capture the specific metabolic derangements at the cellular level, which lead to these changes in ETCO_2._

## 5 Limitations

There were some limitations to this study. First, there was an overall low prevalence of moderate and severe AMS. Previous data from Kilimanjaro summits suggest a 10–20% failure rate due to AMS, however, our summit success limited the power of our study. Second, the device that was used to measure ETCO_2_ is developed to confirm endotracheal tube placement and not portable capnometry. Therefore, the ETCO_2_ measurements were side stream measurement of exhaled carbon dioxide. While this is imperfect, it still provided a consistent, practical, and portable method to measure exhaled carbon dioxide. We attempted to account for this limitation by also measuring baseline ETCO_2_ and change in ETCO_2_. It is worthy to note, that based on our exponential regression model, the measured ETCO_2_ at sea level would be 36 mmHg and this is consistent with measurements performed prior to any of the treks. Finally, the correlations of this study are likely weakened by the comparison of single day hikes starting at different altitudes with a lengthy multi-day trek. In this prospective non-randomized study, we were also unable to account for confounders such as use of medications. Acetazolamide is known to induce physiologic changes, which could affect ETCO_2_ and was used by all hikers who climbed Mt. Kilimanjaro. No hikers on any other trek used medications. Interestingly, the association between ETCO_2_ and altitude was strongest for the shortest trek.

The combined measurements of end tidal carbon dioxide along with respiratory rate may have been a better surrogate marker for minute ventilation that could provide more accurate information on the development of AMS. Studies have shown that the relationship between ETCO_2_ and minute ventilation in non-intubated patients is weak and inconsistent [21]. Future studies should focus on evaluation of ETCO_2_ in association with cellular metabolic changes in patients with High Altitude Cerebral Edema, and High Altitude Pulmonary Edema.

## 6 Conclusions

This is the first study to examine the relationship between real-time ETCO_2_ and AMS. ETCO_2_ was strongly correlated with altitude and moderately correlated with AMS. ETCO_2_ was a better predictor for development of AMS than altitude. An ETCO_2_ measurement of 22 mmHg or lower was 100% sensitive and 60% specific for predicting AMS. ETCO_2_ may be a useful portable modality to predict and diagnose AMS.

## Article summary

Why is this topic important?

Current predictors for altitude related illness are imperfect and do not provide clinical guidance during ascent.

What does this study attempt to show?

This study attempts to demonstrate that portable ETCO_2_ measurements can provide dynamic information during ascent to high altitude that may predict acute mountain sickness.

What are the key findings?

ETCO_2_ was a better predictor for development of acute mountain sickness than altitude. An ETCO_2_ measurement of less than 22 mmHg was 100% sensitive and 60% specific for prediction of acute mountain sickness. Climbers who developed an ETCO_2_ of 22 mmHg or lower had a 25 times higher risk of developing acute mountain sickness.

How is patient care impacted?

In the field of wilderness medicine and austere settings, portable ETCO_2_ can accurately predict who is at high risk for mild and severe acute mountain sickness. Medical professionals can be better equipped to determine when patients would benefit from descent to avoid clinical deterioration.

## Author contribution statement

Josef George Thundiyil, MD, MPH: Conceived and designed the experiments; Performed the experiments; Analyzed and interpreted the data; Wrote the paper.

Alex T Williams, MD; ian Little, MD; Linda Papa, MD: Analyzed and interpreted the data; Wrote the paper.

Margaret Stutsman, MD: Performed the experiments; Analyzed and interpreted the data.

Jay Ladde, MD: Conceived and designed the experiments; Performed the experiments; Contributed reagents, materials, analysis tools or data; Wrote the paper.

## Funding statement

This research did not receive any specific grant from funding agencies in the public, commercial, or not-for-profit sectors.

## Data availability statement

Data will be made available on request.

## Declaration of competing interest

None of the authors have any financial or personal interests which would be perceived as influencing this work.
